# Examining hydrogen peroxide-containing organelles in seaweeds

**DOI:** 10.17912/micropub.biology.001217

**Published:** 2024-11-07

**Authors:** Joseph S. Ramahi, Keiko L. Hokeness, John Gonzales, Jadi Allen, Roman D. P. Marquez, Robin A. A. Rojas, Sergey Ingram, Sara Capponi, Jennifer E. Smith, Brian von Herzen, John Dueber, Zheng-Hui He

**Affiliations:** 1 Department of Biology, San Francisco State University, San Francisco, California, United States; 2 Climate Foundation, Woods Hole, Massachusetts, United States; 3 Accelerated Material Discovery and Cellular Engineering, IBM Research - Almaden, San Jose, California, United States; 4 Marine Biology Research Division, Scripps Institution of Oceanography, University of California, San Diego, San Diego, California, United States; 5 Department of Bioengineering, University of California, Berkeley, Berkeley, California, United States

## Abstract

Seaweeds, particularly the red seaweed *Asparagopsis taxiformis* , produce and sequester bromomethanes, which are known for mitigating methane emissions in ruminants when used as a feed supplement. Bromomethane synthesis requires hydrogen peroxide (H _2_ O _2_ ). We developed a staining assay utilizing 3,3′-diaminobenzidine (DAB) for identifying H _2_ O _2_ in three groups of seaweeds (red, brown, and green), including intensely pigmented species. Our findings indicate the previously identified "gland cell" in *Asparagopsis taxiformis* , responsible for bromoform synthesis and retention, is a specialized large organelle rich in H _2_ O _2_ . Our study introduces an effective survey tool to identify promising seaweed species abundant in bromoform from diverse marine habitats.

**Figure 1. Detection of H 2 O 2 -containing organelles in red, brown, and green seaweeds.**
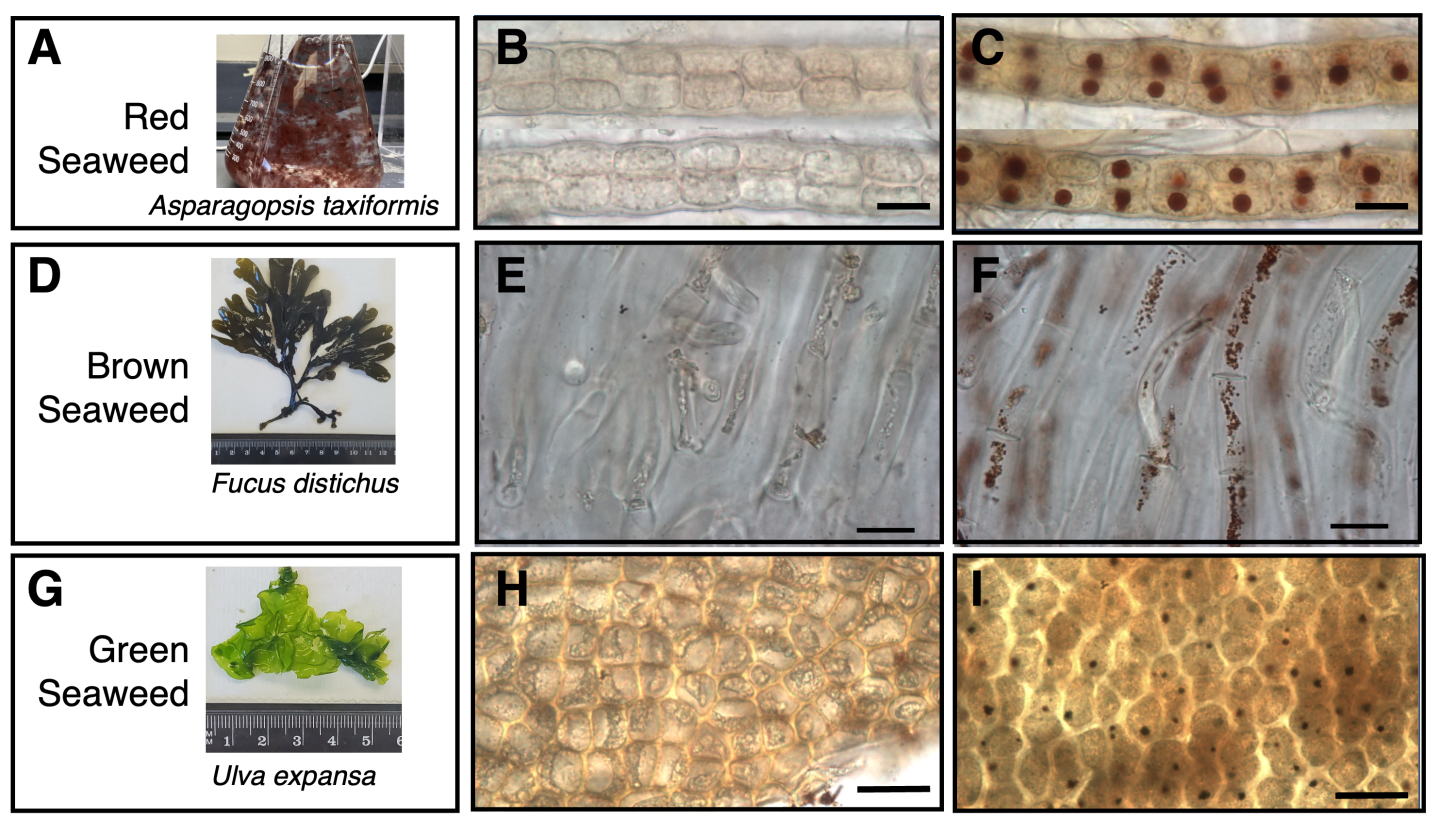
A, D, and G, snapshots of seaweed organisms used in this study - red ( *Asparagopsis taxiformis* ), brown ( *Fucus distichus* ), and green ( *Ulva expansa* ), respectively. *Fucus distichus* and *Ulva expansa* are imaged with a ruler *.* B, E, H, as negative controls, 10 mM ascorbic acid was added to quench out H _2_ O _2_ before DAB staining in *Asparagopsis taxiformis* , *Fucus distichus* , and *Ulva expansa* , respectively. C, F, I, DAB staining to detect H _2_ O _2_ in *Asparagopsis taxiformis* , *Fucus distichus* , and *Ulva expansa* , respectively. Scale bar = 30 µm

## Description

Methane (CH _4_ ) is a significant greenhouse gas (Balcombe et al., 2018) . Considering a 100-year timescale, CH _4_ holds an instantaneous global warming potential that is 120 times higher than that of carbon dioxide (CO _2_ ) (Balcombe et al., 2018) . Methane now contributes to nearly 30% of the total global greenhouse gas effect (Prather and Holmes, 2017). Enteric fermentation in ruminant animals accounts for about 30.4% of global anthropogenic methane emissions, making it the primary anthropogenic source of methane. Anaerobic fermentation by ruminal microbiota is responsible for CH _4_ emission (Roque et al., 2019) . Advanced mitigation technologies are sorely needed to minimize and manage CH _4_ emissions in the livestock industry.

Seaweeds produce various chemical compounds that play important antimicrobial roles (Paul et al., 2006) . Bromomethanes, halogenated organic compounds, are naturally produced in most seaweeds and other algae in large quantities globally (Stemmler et al 2015). These halomethanes are shown to be effective in inhibiting fermentation in ruminant methanogens (Roque et al., 2019, Palangi et al., 2022) . Specifically, bromoform competitively binds and inhibits Coenzyme M, the second to last enzymatic step in the production of methane, resulting in a significant reduction of methane output in vitro and in agricultural ruminants (De Bhowmick et al., 2023) .

In particular, *Asparagopsis taxiformis ( A. taxiformis )* , a tropical to subtropical red seaweed, synthesizes and retains high levels of bromoform, CHBr _3_ . *A. taxiformis* has been successfully used as a feed supplement to reduce methane emissions in ruminants by up to 90% (Roque et al., 2019) . The marine bromomethane biosynthesis (mbb) locus comprises four genes ( *mbb1-4* ) that encode multiple bromoperoxidases (Thapa et al., 2020) . These enzymes catalyze the reaction of bromine, an ion abundantly found in seawater, and hydrogen peroxide to produce bromomethanes. Prior studies have shown that the mbb locus is linked to CHBr _3_ production in *A. taxiformis* and the bromoform compound is retained and concentrated in a structure previously called the “gland cell” (Thapa et al., 2020; Hargrave et al., 2024) .

There is increasing interest in developing large-scale cultivation of *Asparagopsis* for methane mitigation, but this seaweed can be challenging to grow at scale As such, there is a strong interest to identify other bromoform-rich seaweeds across diverse marine habitats that can potentially serve as a feed supplement. Significant production of bromomethanes occurs in other seaweeds including the giant kelp *Macrocystis spp .* , but the retention of these bromomethanes, including bromoform, is far lower than in *Asparagopsis* . Currently, gas chromatography-mass spectrometry (GC-MS) is used to quantify the concentration of bromoform. However, this method doesn't relate spatial information to determine where bromoform is produced in a cost-effective manner that can be performed in seaweeds across a variety of marine habitats. In this study, we use hydrogen peroxide as a proxy to investigate hydrogen peroxide-containing organelles as hydrogen peroxide is required for CHBr _3_ production *in vivo* . In the presence of peroxidase, such as the bromoperoxidase, 3,3'-diaminobenzidine (DAB), a colorless compound, reacts with H _2_ O _2_ to form a dark purple/brown product that can be observed microscopically. DAB has been widely used to detect H _2_ O _2_ -containing peroxisomes in plants (Daudi et al., 2012) . Here, we probe whether other northern California seaweeds have abundant H _2_ O _2_ -containing organelles as potential bromoform biosynthesis sites. To do this, we utilize 3,3'-diaminobenzidine (DAB) stain, predominantly used in terrestrial *Arabidopsis thaliana* , as the output of a dark purple/brown substrate forms upon oxidation by hydrogen peroxide in the presence of peroxidases, such as bromoperoxidase (Daudi et al., 2012) . The biggest challenges of DAB staining in seaweeds are sample thickness and removal of natural pigments.

The hydrogen peroxide content of many seaweeds remains unknown. We are interested in identifying local species that are also high in bromoform production to provide other options for feed additives. We use hydrogen peroxide as a proxy for CHBr _3_ identification in *A. taxiformis* as a survey tool to effectively identify CHBr _3_ in various seaweed species.

Three diverse macroalgae were investigated for the presence of H _2_ O _2_ -containing organelles using DAB staining. Our results demonstrated the presence of diverse H _2_ O _2_ -containing organelles in the three seaweed species. The red seaweed, *A. taxiformis* , was collected in San Diego, isolated and purified in the lab at Scripps Institution of Oceanography and ultimately grown in sterile seawater in a culture flask in the lab at SFSU ( Figure 1A ). The brown seaweed, *Fucus distichus* ( *F. distichus* ), and the green seaweed, *Ulva expansa* ( *U. expansa* ), were locally collected from San Francisco Bay ( Figure 1 D and 1G). DAB is vacuum infiltrated into live seaweed tissue in a similar manner as reported in plants (Daudi et al., 2012) . As controls, all three seaweed tissues were pre-infiltrated with ascorbic acid to quench out H _2_ O _2_ before DAB staining. As shown in Figure 1B, the bead-like pericentral cells of *A. taxiformis* give a clear background after de-staining, indicating the ascorbic acid infiltration successfully removed H _2_ O _2_ in this filamentous seaweed. The brown seaweed, *F. distichus* , is thicker and more pigmented than *A. taxiformis* . Nonetheless, ascorbic acid infiltration effectively removed H _2_ O _2_ in *F. distichus* tissues ( Figure 1E ). As shown in Figure 1C, DAB detected a large round H _2_ O _2_ -containing organelle in most *A. taxiforms* cells. These organelles were previously described as “gland cells” that are known to be involved in bromoform biosynthesis. These H _2_ O _2_ -rich structures can be directly microscopically examined in whole-mounted tissues, and as seen in Figure 1C, are contained in each cell. DAB staining is exclusively detected in these round organelles. Little DAB staining is detected in pericentral cellular space ( Figure 1C ). For the brown seaweed, *F. distichus ,* the DAB-stained tissues are thick and dark and thus incompatible with whole-tissue mount imaging. Cryo-sectioning was used to create thin sections of DAB-stained *F. distichus* tissue. As shown in Figure 1F *,* H _2_ O _2_ -containing organelles in *F. distichus* are detected as small punctates in the cytoplasmic space of the elongated cylindrical cells. The thin thallus of the green seaweed, *U. expansa* , comprises two layers of cells, thus can be microscopically examined without sectioning. DAB staining detected H _2_ O _2_ -containing organelles as individual punctate within the cytoplasm of each cell. These patterns are consistent with the reported peroxisome pattern mapped by YFP-tagged with a peroxisome targeting signal (Blomme et al. 2021) . Our studies with the three diverse macroalgae show that H _2_ O _2_ -containing organelles are highly diverse in their numbers and shapes across taxa. While *A. taxiformis* and *U. expansa* have one centralized H2O2-containing organelle, *F. distichus* displays numerous small ones around the cell.

Altogether, our findings show that our modified DAB staining protocol effectively detects H _2_ O _2_ -containing organelles in macroalgae. Additionally, our findings indicate that the previously described “gland cell” of *A. taxiformis* is a large, specialized H _2_ O _2_ -containing organelle. Our DAB protocol provides a feasible survey tool that can be used to identify H2O2-rich species across all three macroalgae efficiently. This approach is cost-effective to screen for seaweeds that potentially have high bromoform production. Our DAB imaging data suggest that the staining intensity and stained surface area of the H _2_ O _2_ -containing organelles in A. taxiformis are much more than those in *U. expansa* and *F. distichus* . Future studies will compare the actual bromoform concentrations in various macroalgal species to determine if there is a strong relationship between the bromoform concentrations and the sizes/numbers of these H _2_ O _2_ -containing organelles. While H _2_ O _2_ -rich organelles are likely peroxisomes, further experiments are needed to isolate DAB-stained organelles to determine their cellular characteristics.

## Methods

*Seaweed collection and culture:*

*Ulva expansa* and *Fucus distichus* were collected in Tiburon, CA, at the San Francisco State University Estuary and Ocean Science Center during low tide. They were transported and cultured at SFSU. Cold-water seaweed culture was maintained under 14°C, a 16h light / 8h dark photoperiod, and was supplemented with Guillards F2 media (Guillard 1975) in ~35ppt Seawater (Instant Ocean mixed SW) with aeration. *Asparagopsis taxiformis* was collected from San Diego, CA and isolated and purified over the course of several months in Dr. Jennifer Smith's Lab (Scripps Institution of Oceanography of UC San Diego) and sub-cultured at SFSU. Prior to experiments, *Asparagopsis* was cultured at 23°C, 16h light / 8h dark photoperiod, in ~35 ppt Seawater with Guillards F4 media (Instant Ocean mixed SW, supplemented with Germanium Dioxide, filter sterilized and autoclaved) and aeration.

*3,3'-diaminobenzidine (DAB) Stain Procedure:*

DAB Stain was prepared by adding 1 mg of 3,3'-diaminobenzidine (Sigma) per each mL of pH 5 seawater (pH adjusted with 1M HCL). DAB stain solution was mixed overnight and made fresh for each experiment. A sample of *Asparagopsis* / *Ulva* / *Fucus* was removed from the culture, rinsed with deionized water, and dried with a kimwipe. The blade was cut into five-millimeter pieces with a razor blade to allow for better DAB infiltration. These pieces were submerged in 1mg/mL DAB, and placed under a vacuum for ten minutes, the vacuum was released and repeated three times. The DAB was aspirated off with a pipette and deionized water was added to stop the DAB reaction, by diluting and washing away residual reagents (H _2_ O _2_ and DAB) and removing unreacted DAB, thereby reducing the availability of ions that drive the peroxidase-catalyzed reaction. The sample was submerged in pure ethanol and boiled for ten minutes as a fixative and to remove pigment from the tissue. Negative controls were pre-treated with 10 mM ascorbic acid prior to DAB staining. DAB stained & Negative controls were stored in 100% EtOH until mounting.

*Sample embedding and Cryosectioning:*

Samples were mounted in OCT (Optimal Cutting Temperature Compound, Fisher) and submerged in an ethanol dry ice bath until fully frozen. OCT-embedded samples were cryo-sectioned into thirty-micron sections to mount on microscope slides.

*Imaging:*

All samples were brightfield imaged at 20X on a Nikon 80i Microscope in the SFSU Cell & Molecular Imaging Center. Images were collected in QCapture Pro as TIFFs.
